# Genetic Uncertainties Fueling Cholangiocarcinoma Progression

**DOI:** 10.2174/0113892029392506251115053421

**Published:** 2026-02-24

**Authors:** Sunil Kumar Kadiri, Prashant Tiwari

**Affiliations:** 1 Department of Pharmacology, College of Pharmaceutical Sciences, Dayananda Sagar University, Deverakeggahali, Kanakapura Road, Ramanagara Dist. Karnataka - 562112, India

**Keywords:** Intrahepatic cholangiocarcinoma, genomic research, epigenetic alterations, malignant transformation, treatment strategies

## Abstract

Intrahepatic cholangiocarcinoma (iCCA) is a highly aggressive type of liver cancer that is difficult to treat due to its complicated causes. It is typically detected in advanced stages, leaving patients with few treatment options. Recent breakthroughs in genomic research have emphasized the pivotal significance of genetic changes in the initiation and advancement of intrahepatic cholangiocarcinoma (iCCA). The objective of this study was to investigate the genetic relationships that are responsible for the development and advancement of intrahepatic cholangiocarcinoma (iCCA), with a specific focus on crucial mutations, gene expressions, and molecular pathways that contribute to the formation of tumors. By performing a thorough examination of the genome, we have discovered multiple genetic abnormalities that are frequently linked to intrahepatic cholangiocarcinoma (iCCA), such as changes in the *IDH1/2, FGFR2*, and *KRAS* genes. These mutations have been discovered to promote cancer-causing processes by interfering with regular cellular functioning and increasing the likelihood of malignant transformation. Furthermore, the study has examined the influence of epigenetic alterations on gene expression, which enhances the diversity and aggressive characteristics of iCCA. The results have emphasized the significance of genetic profiling in comprehending the molecular pathways that underlie intrahepatic cholangiocarcinoma (iCCA) and highlighting prospective targets for therapy. This review has made an attempt to provide a clear understanding of the genetic characteristics of iCCA, which can lead to the development of tailored treatment strategies. This has the potential to improve outcomes for patients with this challenging condition.

## 1 INTRODUCTION

Cholangiocarcinoma (CCA) is an uncommon and severe cancer originating from the epithelial cells of the bile ducts, which transport bile from the liver to the gallbladder and small intestine [1]. The disease is marked by a difficult diagnosis, frequently identified in advanced stages, and typically has a dismal prognosis. The tumor can arise in any segment of the biliary route, resulting in specific clinical symptoms dependent on its location. Intrahepatic cholangiocarcinomas are occasionally categorized as a form of liver cancer [2-4]. Perihilar and distal cholangiocarcinomas are sometimes known as extrahepatic bile duct malignancies. Histologically, about 90% of cholangiocarcinomas are classified as adenocarcinomas [5]. They may range from well-differentiated to undifferentiated tumors. Cholangiocarcinomas are frequently enclosed in a fibrotic or desmoplastic tissue response, complicating the differentiation between well-differentiated tumors and typical reactive epithelium [6]. Cholangiocarcinoma is an uncommon malignancy, impacting around 8,000 individuals annually in the United States. It predominantly occurs in elderly persons, with peak prevalence at approximately 70 years of age. Cholangiocarcinoma exhibits greater prevalence in Southeast Asia, where it is linked to persistent liver fluke infections [7-10].

Cholangiocarcinoma, a neoplasm originating from the bile ducts, is categorized according to its anatomical site, histological properties, and molecular attributes. The principal categories consist of the following:

### 1.1 Anatomical Categorization

The anatomical categories of cholangiocarcinoma are provided as follows: Intrahepatic cholangiocarcinoma (iCCA): It arises from the bile ducts located within the liver.

Perihilar cholangiocarcinoma (pCCA): It arises at the confluence of the left and right hepatic ducts, sometimes referred to as Klatskin tumors.

Distal cholangiocarcinoma (dCCA): It originates from the bile ducts external to the liver, next to the small intestine [11].

### 1.2 Histological Categorization

The histological categorization of the disease is described as follows:

Adenocarcinoma: It is the predominant form, characterized by glandular features or mucin-secreting cells.

**Fig. (1):**
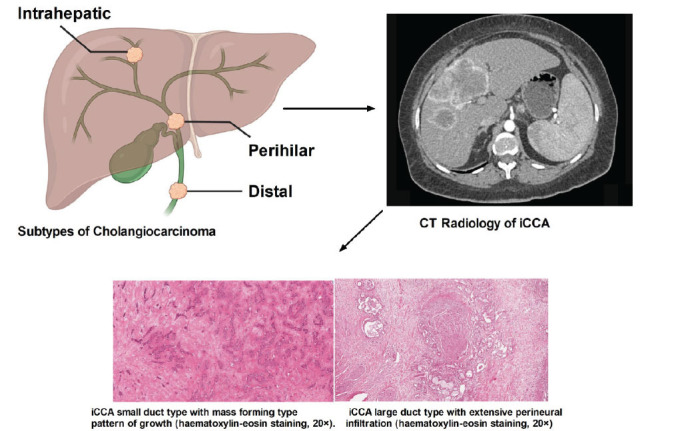
Anatomical and histological characterization of intrahepatic cholangiocarcinoma.

Other variations: These include squamous cell carcinoma, adenosquamous carcinoma, and sarcomatoid variations [12-14] (Fig. **1**).

### 1.3 Molecular and Genetic Classification

Cholangiocarcinomas can be categorized according to their molecular profiles, which may inform targeted therapy [15]. Illustrations encompass *IDH1* and *IDH2* mutations that are prevalent in intrahepatic cholangiocarcinoma, *FGFR2* fusions, mutations in the *KRAS* gene and *BRAF* mutations [16].

### 1.4 TNM Staging

The dimensions and scope of the primary neoplasm are classified under tumor (T), which describes the size and extent of the tumor. The involvement of regional lymph nodes is categorized under node (N), indicating the presence or absence of lymph node metastasis. Metastasis (M) refers to the dissemination of cancer cells to distant organs, assessing whether the cancer has spread beyond the original site. Every classification provides an insight into prognosis, therapeutic approach, and possible reaction to specific treatments [17].

Intrahepatic cholangiocarcinoma (ICC) is an uncommon, yet increasingly identified primary liver malignancy, representing 10-20% of all cholangiocarcinomas [18]. A systematic review and meta-analysis of data from various countries indicated a global increase in the incidence rates of ICC and extrahepatic cholangiocarcinoma (ECC) over the last two decades. Significant increases were observed in countries with historically low incidence rates, including various European nations and Australia. In contrast, countries, such as Thailand, demonstrated a nonsignificant reduction in ICC incidence [19]. Numerous meta-analyses have pinpointed significant risk factors for CCA. A study indicated that obesity correlates with an increased risk of developing CCA. Furthermore, viral hepatitis is recognized as a notable risk factor, supported by a meta-analysis demonstrating an elevated risk of CCA in individuals with chronic hepatitis B or C infections [20]. Extensive research has been conducted on prognostic factors influencing overall survival in patients with resected hilar cholangiocarcinoma. A meta-analysis of 24 studies encompassing 4,599 patients revealed several significant prognostic indicators: age, tumor category (T stage), lymph node involvement, microvascular invasion, perineural invasion, and tumor differentiation. These studies emphasize the global burden of CCA and the necessity of identifying modifiable risk factors and prognostic indicators to enhance patient outcomes. The global prevalence has increased over the past few decades, especially in Southeast Asia, where liver fluke infections, such as *Opisthorchis viverrini* and *Clonorchis sinensis,* are endemic and recognized as risk factors. Additional significant risk factors for intrahepatic cholangiocarcinoma (ICC) encompass chronic liver diseases, including cirrhosis, hepatitis B and C infections, and non-alcoholic fatty liver disease (NAFLD). Conditions that induce chronic biliary inflammation, including primary sclerosing cholangitis (PSC), choledochal cysts, and hepatolithiasis, further elevate the risk. Moreover, environmental exposures to toxins, such as thorotrast, and genetic alterations in *IDH1*, *IDH2,* and *FGFR2*, have been associated with its development. The diagnosis of intrahepatic cholangiocarcinoma (ICC) generally necessitates a combination of imaging studies, laboratory analyses, and histopathological verification. Preliminary imaging techniques, such as ultrasound, computed tomography (CT), or contrast-enhanced magnetic resonance imaging (MRI), are employed to assess the tumor's features and scope [21]. The MRI with MRCP (magnetic resonance cholangiopancreatography) is especially effective in elucidating the biliary tree. Tumor markers, such as increased serum CA 19-9 and carcinoembryonic antigen (CEA), can assist in diagnosis, but they are not specific. A conclusive diagnosis typically necessitates a biopsy, frequently conducted with imaging guidance [22-25]. Clinical staging adheres to the TNM (tumor-node-metastasis) approach, which evaluates tumor dimensions and extent (T), lymphatic node involvement (N), and distant metastasis (M). ICC is often classified as localized, locally progressed (including nearby organs or blood vessels), or metastatic, with prognosis being worse as the stage progresses [26].

## 2 MOLECULAR AND GENETIC ALTERATIONS IN ICC

### 2.1 Common Genetic Mutations in Intrahepatic Cholangiocarcinoma (*e.g*., IDH1/2, FGFR2, BAP1, TP53)

**Fig. (2):**
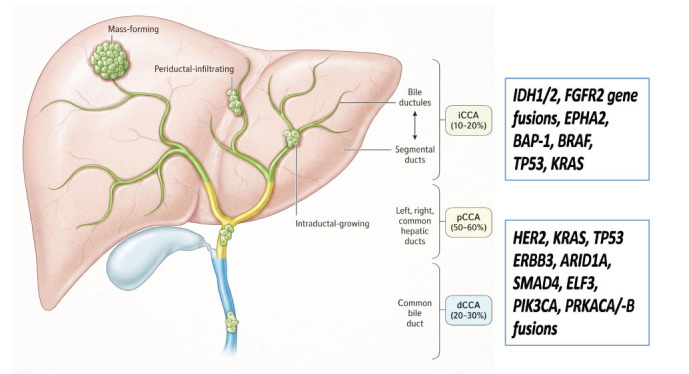
Molecular modifications of cholangiocarcinoma (CCA) subtypes. AT-Rich interaction domain 1A (AIRD1A); BRCA1-associated protein 1 (BAP-1); Proto-oncogene B-Raf (BRAF); Ductus hepaticus communis (DHC); Human epidermal growth factor receptor 2 (Her2); E74-like ETS transcription factor 3 (ELF3); Ephrin type-A receptor 2 (EPHA2); Erb-B2 receptor tyrosine kinase 3 (ERBB3); Fibroblast growth factor receptor gene 2 (FGFR 2), Isocitrate dehydrogenase 1 and 2 (IDH1/2); Kirsten rat sarcoma (KRAS); Phosphatidylinositol-4,5-bisphosphate 3-kinase catalytic subunit alpha (PIK3CA); Protein kinase CAMP-activated catalytic subunit-alpha/-beta (PRKACA/-B); SMAD family member 4 (SMAD4); Tumor protein P53 (TP53).

Intrahepatic cholangiocarcinoma, a malignancy originating from the bile ducts in the liver, displays specific genetic alterations that influence its development, progression, and treatment response [27]. Progress in genomic profiling has shown multiple recurring mutations in intrahepatic cholangiocarcinoma (iCCA), with *IDH1/2, FGFR2, BAP1*, and *TP53* being the most prevalent (Fig. **2**) [109]. *IDH1/2* mutations are present in around 15-20% of intrahepatic cholangiocarcinoma patients [28]. These mutations lead to the synthesis of an oncometabolite, 2-hydroxyglutarate, which interferes with normal cellular metabolism and epigenetic control, hence facilitating cancer. Targeted inhibitors of mutant *IDH* enzymes, including ivosidenib, have demonstrated potential in the treatment of *IDH*-mutant *iCCA. FGFR2* fusions and rearrangements occur in approximately 10-15% of iCCA patients [29]. These modifications result in the constitutive activation of the *FGFR2* signaling pathway, promoting unregulated cell proliferation and survival. *FGFR* inhibitors, such as pemigatinib, have received approval for *FGFR2* fusion-positive iCCA, highlighting the significance of molecular testing for tailored treatment. Mutations in *BAP1*, a tumor suppressor gene implicated in chromatin remodeling and DNA repair, are present in a subset of iCCA patients. The loss of *BAP1* function correlates with genomic instability and heightened vulnerability to malignant transformation [30]. *TP53*, a gene commonly altered in numerous malignancies, is essential for the regulation of the cell cycle and apoptosis. *TP53* mutations are associated with more aggressive tumor characteristics and a worse prognosis in iCCA patients [31]. Comprehending these genetic modifications has profoundly impacted the formulation of individualized therapeutic approaches. Molecular analysis of iCCA tumors facilitates the identification of actionable mutations, permitting tailored medicines that enhance patient outcomes and provide new optimism in the management of this complex malignancy.

### 2.2 Chromosomal Abnormalities and Gene Amplifications

Chromosomal abnormalities and gene amplifications significantly contribute to the development and progression of intrahepatic cholangiocarcinoma (iCCA). These genetic modifications interfere with normal cellular functions, facilitating tumor proliferation, persistence, and metastasis. Frequent chromosomal anomalies in iCCA encompass both amplifications and deletions of particular chromosomal regions [32]. Chromosomal gains are frequently detected at 1q, 7p, 8q, and 17q locus genes. These areas frequently include oncogenes whose overexpression can induce cancer. In contrast, chromosomal losses frequently occur on 3p, 6q, 9p, and 14q, resulting in the deletion or inactivation of tumor suppressor genes, which facilitates malignant transformation and progression. Gene amplifications augment oncogenic signaling pathways in iCCA. Prominent amplified genes encompass *ERBB2 (HER2), MET*, and *MDM2*. Amplification of *ERBB2 (HER2)* results in excessive activation of the *HER2* signaling pathway, facilitating cellular proliferation and survival. Likewise, MET amplification augments the hepatocyte growth factor (HGF) pathway, facilitating tumor proliferation and metastasis. *HER2* and *MET* amplifications represent viable therapeutic targets, with *HER2*-targeted medicines and *MET* inhibitors demonstrating potential in clinical studies [33]. *MDM2* amplification impedes the tumor suppressor function of *p53*, leading to less apoptosis and enhanced cell proliferation. Furthermore, *CCND1* amplification results in cyclin D1 overexpression, which impairs cell cycle regulation and fosters unregulated cellular proliferation [34]. These genetic modifications enhance our comprehension of iCCA pathophysiology and underscore potential biomarkers for targeted therapeutics, facilitating more individualized treatment strategies in iCCA management.

### 2.3 Somatic *vs*. Germline Mutations in ICC

In iCCA, somatic and germline mutations contribute to disease development and progression, although with distinct origins and implications [35, 36]. Somatic mutations arise in non-germline cells and are acquired during an individual's life, frequently as a result of environmental influences, persistent inflammation, or random errors in DNA replication. These mutations are not inherited nor transmitted to progeny [37]. Frequent somatic mutations in iCCA encompass modifications in *IDH1/2*, *FGFR2* fusions, *BAP1*, and *TP53*, all of which are susceptible to targeted precision therapy. Conversely, germline mutations are hereditary genetic modifications found in all bodily cells, including gametes, and can be transmitted to subsequent generations. Germline mutations linked to iCCA are infrequent but may affect tumor suppressor genes, like *BRCA1/2* or *MLH1*, associating iCCA with hereditary cancer disorders. Identifying germline mutations is crucial for familial risk evaluation, whereas somatic mutations guide customized therapeutic approaches.

### 2.4 Role of Epigenetic Modifications in Cholangiocarcinoma Progression

Epigenetic changes are essential in the advancement of CCA by modulating gene expression without changing the DNA sequence. Principal epigenetic mechanisms encompass DNA methylation, histone changes, and non-coding RNAs. Aberrant DNA methylation frequently results in the silencing of tumor suppressor genes, including *p16INK4a* and *RASSF1A*, hence facilitating unchecked cellular proliferation and evasion of apoptosis. Histone changes, such as acetylation and methylation, modify chromatin architecture, influencing the transcription of genes pertinent to cell cycle regulation and metastasis. The overexpression of histone-modifying enzymes, such as *EZH2*, is associated with unfavorable outcomes in CCA. Furthermore, non-coding RNAs, including microRNAs (miRNAs) and long non-coding RNAs (lncRNAs), modulate gene expression *via* governing mRNA stability and translation. The dysregulation of particular miRNAs, such as miR-21, facilitates tumor proliferation, invasion, and resistance to chemotherapy. The reversible nature of these epigenetic alterations presents opportunities for tailored therapeutics employing epigenetic inhibitors to impede CCA progression and enhance patient outcomes. Repetitive and satellite DNA elements are essential for genomic integrity, and their dysregulation is frequently observed in numerous malignancies. These components, such as long interspersed nuclear elements (LINEs) and satellite DNA, frequently encounter hypomethylation, resulting in genomic instability, modified gene expression, and heightened tumor aggressiveness [38]. In CCA, research indicates that *CpG* island hypermethylation occurs early in tumor development, but repetitive DNA hypomethylation is noted in later stages. Methylation levels of *LINE-1* and satellite 2 (*SAT2*) are markedly reduced in CCA relative to normal bile duct tissues, signifying a lack of epigenetic regulation. This hypomethylation may lead to chromosomal instability and tumor progression. Comparable patterns of repeated element dysregulation have been identified in other malignancies, including prostate cancer, where hypermethylation of satellite DNA has been associated with tumor growth [39]. Moreover, microsatellite instability (MSI), a condition linked to mismatch repair deficiency, has been related to multiple cancers, including colorectal and gastric malignancies. The findings indicate that epigenetic modifications in repeated sequences may function as potential biomarkers for cancer diagnosis and prognosis.

### 2.5 Genomic and Transcriptomic Insights into Cholangiocarcinoma: A Comparative Analysis with Osteosarcoma

CCA is a very diverse and aggressive neoplasm with restricted treatment alternatives. Recent genomic and transcriptomic investigations have yielded significant insights into its molecular architecture. A study performed a multi-omics characterization of CCA, incorporating whole-genome sequencing (WGS), whole-exome sequencing (WES), RNA sequencing, and whole-genome bisulfite sequencing (WGBS) to categorize CCA into four unique molecular subtypes. These subgroups demonstrated distinct tumor microenvironment attributes and varying responses to immune checkpoint blockade therapy [40]. A separate study investigated the clonal evolution of CCA by whole-exome and transcriptome sequencing, demonstrating the impact of genomic mutations on transcriptomic changes and tumor progression. This work emphasized the relationship between somatic mutations and gene expression, offering insights into tumor heterogeneity. In comparing CCA with osteosarcoma, both cancers display intricate genomic landscapes; nevertheless, osteosarcoma is distinguished by significant chromosomal rearrangements and a pronounced mutation burden [41]. CCA research concentrates on interactions within the immunological microenvironment and epigenetic modifications, whereas osteosarcoma studies frequently highlight genomic instability and abnormalities in tumor suppressor genes. A transcriptome investigation found oxidative stress-related molecular subgroups in CCA, which may function as prognostic indicators. This research work employed data from The Cancer Genome Atlas (TCGA) to construct a predictive model centered on oxidative stress-related genes. The findings indicated that multi-omics methodologies are essential for comprehending CCA and formulating targeted treatments.

## 3 SIGNALING PATHWAYS INVOLVED IN ICC PROGRESSION

### 3.1 Notch, Wnt, and Hedgehog Signaling Pathways

**Fig. (3):**
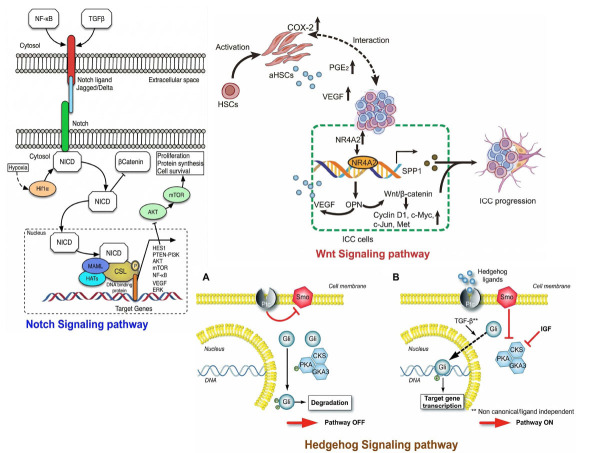
Notch, Wnt, and Hedgehog signaling pathways involved in ICC progression.

The Notch, Wnt, and Hedgehog signaling pathways are essential regulators of embryonic development, tissue homeostasis, and cellular fate determination. Abnormal activation of these pathways significantly contributes to the development and progression of different malignancies, including iCCA.The Notch signaling system is essential for cell differentiation, proliferation, and death. In iCCA, the overactivation of Notch receptors, especially Notch1 and Notch2, has been associated with enhanced tumor proliferation, survival, and resistance to apoptosis. Notch signaling additionally enhances epithelial-to-mesenchymal transition (EMT), hence promoting tumor invasiveness and metastasis. Preclinical studies indicate that the inhibition of Notch signaling may impede tumor proliferation, positioning it as a viable therapeutic target [42]. The *Wnt/β-catenin* signaling system is a vital modulator of cellular proliferation and differentiation. In iCCA, deregulation of this system, frequently due to mutations in β-catenin or other pathway constituents, results in the accumulation of β-catenin within the nucleus. This buildup triggers oncogenic transcriptional pathways that facilitate tumor growth, invasion, and chemoresistance [43]. Aberrant *Wnt* signaling also plays a role in sustaining cancer stem cells, which are associated with tumor recurrence and resistance to standard therapy. The Hedgehog signaling system, crucial for embryonic development, is often dormant in adult tissues but may be triggered in malignancies, such as iCCA [44-48] (Fig. **3**). In this environment, Hedgehog signaling facilitates desmoplasia (the creation of fibrotic stroma), hence aiding tumor proliferation, invasion, and metastasis. It also improves the tumor microenvironment's resistance to treatment. Inhibiting Hedgehog signaling with particular agents has demonstrated potential in preclinical investigations for diminishing tumor proliferation and enhancing treatment results. These signaling pathways are essential in the pathogenesis of cholangiocarcinoma, and their blockage offers significant opportunities for innovative, tailored therapeutics to enhance patient outcomes in iCCA [49].

### 3.2 PI3K/AKT/mTOR Pathway and its Role in ICC

The *PI3K/AKT/mTOR* signaling pathway is a vital modulator of cellular growth, proliferation, metabolism, and survival [50]. Its dysregulation significantly contributes to the development and progression of iCCA.The initiation of this pathway generally commences with the activation of *PI3K* (phosphoinositide 3-kinase) by growth factors that attach to receptor tyrosine kinases (RTKs), like *EGFR* or *FGFR*. The activation of *PI3K* results in the synthesis of PIP3, which subsequently recruits and activates *AKT*, a serine/threonine kinase. *AKT* subsequently phosphorylates other downstream targets, including mTOR (mechanistic target of rapamycin), which is a crucial regulator of protein synthesis and cellular proliferation [51-54]. In iCCA, mutations or amplifications in upstream *RTKs, PI3K* subunits, or the loss of tumor suppressors, such as *PTEN*, which negatively control this pathway, lead to the constitutive activation of *PI3K/AKT/mTOR* signaling. This facilitates unregulated cellular proliferation, persistence, angiogenesis, and evasion of apoptosis, hence advancing tumor development and chemoresistance. Inhibiting this system with agents, like *mTOR* inhibitors (*e.g*., everolimus) or *PI3K/AKT* inhibitors, has demonstrated promise in preclinical research. The intricacy and redundancy of the route frequently result in resistance, underscoring the necessity for combination medicines to enhance treatment outcomes in iCCA [55].

### 3.3 Role of FGFR and Other Growth Factor Receptors in the Tumor Microenvironment

Fibroblast growth factor receptors (FGFRs), along with other growth factor receptors, including EGFR and VEGFR, are crucial in the tumor microenvironment (TME) of iCCA. FGFRs, especially *FGFR2*, are frequently overexpressed or altered in iCCA, resulting in the constitutive activation of downstream signaling pathways that enhance tumor cell proliferation, survival, and angiogenesis [56]. *FGFR2* fusions or amplifications are notably important in a subset of iCCA patients, facilitating tumor growth and establishing *FGFR2* as a target for precision therapeutics. Growth factor receptors, such as EGFR and VEGFR, play a role in the tumor microenvironment by facilitating cellular responses to external stimuli. Activation of EGFR accelerates tumor proliferation and metastasis, whereas VEGFR facilitates angiogenesis, supplying vital nutrients and oxygen to the expanding tumor. The intricate interactions among these receptors in the tumor microenvironment foster a pro-tumorigenic milieu, facilitating tumor proliferation, invasion, and therapeutic resistance. Targeting these receptors presents therapeutic prospects in iCCA [57].

### 3.4 Crosstalk between Signaling Pathways Influencing Tumor Growth

The interaction between signaling pathways is essential in modulating tumor development and advancement. In iCCA, pathways, such as PI3K/AKT/mTOR, Notch, Wnt, and Hedgehog, interact to facilitate carcinogenesis [58]. Notch signaling can augment Wnt pathway activation, facilitating epithelial-to-mesenchymal transition (EMT) and metastasis. Likewise, PI3K/AKT/mTOR signaling can influence Hedgehog and *FGFR* pathways, facilitating cell survival and angiogenesis. This complex interaction promotes a highly adaptable tumor microenvironment, facilitating cancer cell proliferation, evasion of apoptosis, and resistance to therapies. Simultaneously targeting various pathways may enhance therapeutic effects in iCCA [59].

## 4 ROLE OF TUMOR MICROENVIRONMENT IN GENETIC PROGRESSION

### 4.1 Interaction between Tumor Cells and Surrounding Stroma

The interaction between tumor cells and the adjacent stroma is essential in facilitating tumor growth, notably in iCCA [60-64]. The TME, consisting of stromal cells, extracellular matrix (ECM), immune cells, and blood arteries, actively facilitates tumor growth and spread. Tumor cells release growth factors, cytokines, and matrix metalloproteinases (MMPs) that alter the extracellular matrix (ECM), facilitating cell motility, invasion, and angiogenesis. In iCCA, the stroma undergoes desmoplasia, with fibroblasts, endothelial cells, and immune cells participating in the development of a fibrotic tumor microenvironment [65]. This modified stroma creates an immunosuppressive milieu, constraining anti-tumor immune responses and facilitating cancer cell persistence. Cancer-associated fibroblasts (CAFs) and endothelial cells secrete substances, such as TGF-β and VEGF, which promote fibrosis and angiogenesis. The interaction between tumor cells and the stroma promotes tumor cell invasion, chemoresistance, and metastasis, highlighting the stroma's significance in iCCA pathophysiology and as a potential therapeutic target [66].

### 4.2 Contribution of Immune Cells and Inflammatory Response

Immune cells and the inflammatory response play a crucial role in the advancement of iCCA. Chronic liver inflammation, frequently resulting from illnesses, such as cholangitis or liver cirrhosis, establishes an immunosuppressive tumor microenvironment [67]. Tumor-associated macrophages (TAMs), regulatory T cells (Tregs), and myeloid-derived suppressor cells (MDSCs) facilitate tumor proliferation and metastasis by the secretion of cytokines, such as TGF-β and IL-10, which inhibit immune responses and bolster tumor cell viability. Moreover, neutrophils and dendritic cells can promote cancer cell invasion and angiogenesis, hence advancing iCCA progression [68]. Modulating the inflammatory response may enhance treatment effectiveness and mitigate immune evasion.

### 4.3 Hypoxia-induced Genetic Changes

Hypoxia, a prevalent characteristic of malignancies, triggers genetic alterations that accelerate cancer advancement, particularly in iCCA [69]. Under hypoxic conditions, hypoxia-inducible factors (HIFs) are activated, enhancing the expression of genes associated with angiogenesis, such as VEGF, and glycolysis, such as LDHA, to facilitate tumor survival. Hypoxia induces mutations and epigenetic modifications, facilitating tumor cell invasion and conferring resistance to chemotherapy [70-73]. Moreover, it aids in the selection of cancer stem cells, which exhibit greater resistance to hypoxia-induced stress. Hypoxia-induced genomic alterations engender an aggressive tumor phenotype, facilitating metastasis and resistance to therapy in iCCA [74].

### 4.4 Impact of Angiogenesis on Tumor Development

Angiogenesis, the creation of new blood vessels, is essential in tumor progression, particularly in iCCA. Tumors release pro-angiogenic substances, such as VEGF, which stimulate endothelial cells to generate new blood vessels, thereby providing oxygen and nutrition to expanding tumor masses [75]. This vascular network also enables metastasis by offering a pathway for cancer cells to infiltrate the bloodstream. Angiogenesis facilitates tumor proliferation, viability, and therapeutic resistance by improving nutrient supply and waste elimination. Inhibiting angiogenesis has emerged as a promising therapeutic approach in iCCA, with the goal of depriving tumors of nutrients and diminishing their metastatic potential.

## 5 EMERGING GENETIC BIOMARKERS FOR ICC DIAGNOSIS AND PROGNOSIS

### 5.1 Identifying Potential Biomarkers for Early Detection

Identifying new biomarkers for the early diagnosis of iCCA is essential for enhancing patient outcomes, given that the illness is frequently detected at advanced stages [76]. Biomarkers may be identified in blood, bile, or tissue samples, and can encompass genetic, epigenetic, proteomic, and metabolomic modifications. Prevalent genetic alterations, including *IDH1/2, FGFR2* fusions, and *BAP1* mutations, have been recognized as prospective biomarkers [77]. Furthermore, increased serum markers, such as CA19-9 and CEA, are frequently correlated with iCCA; however, they lack specificity. Epigenetic modifications, including DNA methylation patterns in tumor suppressor genes, such as RASSF1A, and altered expression of microRNAs (*e.g*., miR-21), are under investigation as potential early diagnostic markers [78]. Proteomic analysis has revealed anomalous proteins associated with tumor proliferation and immune response. Liquid biopsies that examine circulating tumor DNA (ctDNA) and exosomes present promising non-invasive methods for early detection [79]. The integration of various biomarkers may improve diagnostic precision and enable earlier action.

### 5.2 Genetic Markers Associated with Prognosis and Survival Rates

Genetic indicators are crucial for predicting prognosis and survival rates in iCCA, providing insights into tumor dynamics and possible therapeutic responses. Mutations in genes, such as *TP53* and *KRAS*, correlate with unfavorable prognosis, aggressive tumor proliferation, and reduced overall survival. *TP53* mutations impair normal cell cycle regulation, resulting in unregulated proliferation, whereas *KRAS* mutations stimulate oncogenic signaling, facilitating tumor advancement and therapeutic resistance. In contrast, specific genetic modifications are associated with improved results. *IDH1/2* mutations, found in a portion of iCCA patients, correlate with reduced tumor development and enhanced survival, perhaps attributable to the accessibility of targeted therapy, such as *IDH* inhibitors [80]. Likewise, *FGFR2* fusions signify a more favorable prognosis, as they demonstrate a positive response to *FGFR*-targeted therapy. Additional indicators, including *BAP1* mutations, are associated with chromatin remodeling abnormalities and exhibit variable prognostic significance contingent upon the tumor setting. Furthermore, *ARID1A* mutations, which play a role in chromatin remodeling, are linked to unfavorable consequences in certain instances [81]. The incorporation of genetic markers into clinical practice enhances prognostication and facilitates the customization of treatment methods, hence enhancing survival rates and quality of life for iCCA patients.

### 5.3 Liquid Biopsy and Circulating Tumor DNA (ctDNA) for Monitoring Disease Progression

**Fig. (4):**
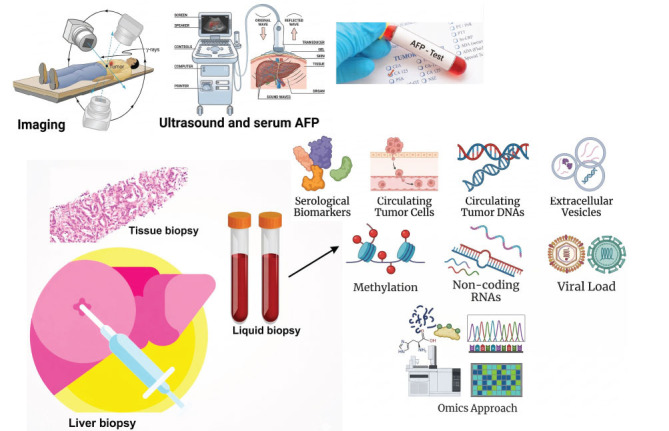
Biomarkers for intrahepatic cholangiocarcinoma diagnosis.

Liquid biopsy and analysis of circulating tumor DNA (ctDNA) have emerged as potent, non-invasive methods for tracking disease development in iCCA. Liquid biopsies entail the examination of blood samples to identify circulating tumor DNA (ctDNA), which comprises fragmented DNA released into the circulation by apoptotic cancer cells [82]. This technique facilitates real-time evaluation of tumor dynamics, presenting numerous benefits compared to conventional tissue biopsies, including less patient risk, reproducibility, and the capacity to capture tumor heterogeneity. ctDNA analysis can identify particular genetic mutations linked to iCCA, including *IDH1/2* mutations, *FGFR2* fusions, and *TP53* mutations, facilitating tailored treatment strategies [83]. Tracking ctDNA levels longitudinally aids in evaluating treatment efficacy, identifying minimum residual disease, and recognizing early indicators of recurrence or therapeutic resistance. A reduction in ctDNA levels may signify effective treatment, whereas an increase in ctDNA levels could indicate disease development prior to the manifestation of clinical signs. Additionally, ctDNA profiling can identify novel genetic variants that exhibit resistance to targeted medicines, facilitating prompt modifications in treatment approaches. With technological advancements, liquid biopsies are emerging as a crucial instrument for dynamic disease surveillance, providing optimism for enhanced outcomes *via* early intervention and tailored treatment in iCCA (Fig. **4**) [84].

## 6 TARGETED THERAPIES AND GENETIC APPROACHES

### 6.1 Current Targeted Therapies Based on Genetic Mutations (*e.g*., FGFR Inhibitors, IDH Inhibitors)

Targeted medicines based on distinct genetic alterations have transformed the treatment paradigm of iCCA, providing more individualized and efficacious alternatives for patients. *FGFR2* fusions and *IDH1/2* mutations represent some of the most promising targets, resulting in the development of particular inhibitors that enhance clinical results. *FGFR* inhibitors specifically target changes in fibroblast growth factor receptor (*FGFR*), notably *FGFR2* fusions, which occur in roughly 10-15% of iCCA patients [85]. These fusions lead to the constitutive activation of the *FGFR* signaling pathway, facilitating unregulated cell proliferation. Pemigatinib and infigratinib are *FGFR* inhibitors authorized for the treatment of iCCA patients with *FGFR2* fusions or rearrangements [86]. Clinical trials have demonstrated that these medications enhance progression-free survival and overall response rates, providing a feasible therapy alternative for this patient population [87]. *IDH* inhibitors represent a distinct category of targeted therapy employed in iCCA patients with *IDH1* or *IDH2* mutations, found in 15-20% of cases. These mutations lead to the synthesis of the oncometabolite 2-hydroxyglutarate, which facilitates carcinogenesis *via* epigenetic alterations. Ivosidenib, an *IDH1* inhibitor, has demonstrated effectiveness in enhancing progression-free survival in individuals with *IDH1*-mutant iCCA. In addition to *FGFR* and *IDH* inhibitors, various novel targeted treatments are under investigation [88]. BRAF mutations, albeit infrequent, may exhibit responsiveness to *BRAF* inhibitors, whereas drugs targeting the *PI3K/AKT/mTOR* pathway are under investigation for cancers with anomalies in this signaling cascade. Moreover, immune checkpoint drugs that target PD-1/PD-L1 are being integrated with targeted therapy to augment anti-tumor responses. The advancement of tailored medicines derived from genetic mutations represents a substantial progress in the treatment of iCCA, enhancing survival and quality of life for patients with certain molecular changes [89].

### 6.2 Precision Medicine in ICC: Tailoring Treatments to Genetic Profiles

Precision medicine in iCCA is a revolutionary strategy in cancer therapy, emphasizing the customization of medicines according to individual genetic and molecular characteristics. In contrast to conventional uniform approaches, precision medicine utilizes genomic profiling to detect mutations and changes that promote tumor proliferation, allowing doctors to choose tailored medicines for more effective and individualized treatment [90]. Principal genetic modifications in iCCA encompass *FGFR2* fusions, *IDH1*/2 mutations, BRAF mutations, and abnormalities in the PI3K/AKT/mTOR signaling pathway. Patients with *FGFR2* fusions derive advantages from FGFR inhibitors, like pemigatinib and infigratinib, which have demonstrated substantial therapeutic success in enhancing progression-free survival. *IDH1* mutations, found in about 15-20% of iCCA cases, can be effectively targeted with ivosidenib, an *IDH1* inhibitor that has shown promising results in clinical trials. Additionally, BRAF mutations can be addressed with BRAF inhibitors, while immune checkpoint drugs that target PD-1/PD-L1 are efficacious in patients exhibiting microsatellite instability-high (MSI-H) or elevated tumor mutational burden (TMB) [91]. Precision medicine facilitates the identification of *KRAS* and *TP53* mutations, which, despite being associated with a worse prognosis, offer essential insights for treatment strategy formulation. Innovative tools, like liquid biopsies and circulating tumor DNA (ctDNA) analysis, augment precision medicine by facilitating real-time observation of tumor progression, therapeutic response, and the development of resistance mutations. This adaptive methodology facilitates prompt modifications in treatment, enhancing patient results. Precision medicine in iCCA presents the possibility for enhanced survival rates, less treatment toxicity, and more individualized care, signifying a notable progression in the therapy of this complex cancer.

### 6.3 Immunotherapy and the Role of Genetic Changes in Predicting the Treatment Response

Immunotherapy has surfaced as a promising intervention for iCCA, especially with immune checkpoint inhibitors that target PD-1, PD-L1, and CTLA-4. The efficacy of immunotherapy differs among patients, with genetic alterations significantly influencing treatment response prediction [92]. Genetic modifications, including microsatellite instability-high (MSI-H) and deficient mismatch repair (dMMR), correlate with improved responses to checkpoint inhibitors, since they result in elevated tumor mutational burden (TMB) and augmented neoantigen presentation, hence boosting immune recognition [93]. Moreover, tumors exhibiting elevated PD-L1 expression are more prone to respond to PD-1/PD-L1 inhibitors. Conversely, mutations in genes, such as *TP53* and *KRAS*, are frequently associated with unfavorable immunotherapy results, as they promote an immunosuppressive tumor microenvironment. The identification of genetic markers by genomic profiling facilitates tailored immunotherapy strategies, enhancing treatment efficacy and improving survival results in iCCA patients [94]. Immunotherapy is increasingly recognized as a viable treatment approach for biliary tract cancers (BTCs), particularly iCCA, which has historically shown resistance to standard therapies. Due to the highly immunosuppressive tumor microenvironment (TME) marked by tumor-associated macrophages (TAMs), regulatory T cells (Tregs), and myeloid-derived suppressor cells (MDSCs), checkpoint inhibitors have emerged as noteworthy therapeutic agents. Immune checkpoint inhibitors (ICIs) that target PD-1 (nivolumab, pembrolizumab) and *PD-L1* (atezolizumab, durvalumab) are currently undergoing clinical trials. Results indicate enhanced response rates, especially in tumors characterized by microsatellite instability-high (MSI-H) or mismatch repair-deficiency (dMMR) profiles. Most iCCA cases are categorized as immune cold, indicating a deficiency in significant immune infiltration and a potential ineffectiveness of single-agent checkpoint blockade. Combination strategies have been developed to enhance the effectiveness of immune checkpoint inhibitors (ICIs). These strategies include the use of checkpoint inhibitors in conjunction with chemotherapy agents, such as gemcitabine and cisplatin, targeted therapies, like *FGFR* and *IDH1* inhibitors, and innovative approaches, such as adoptive cell therapies (*e.g*., CAR-T cells targeting tumor-specific antigens), as well as personalized cancer vaccines aimed at improving tumor antigen recognition. Furthermore, novel approaches, like cytokine-based immunotherapies, aimed at counteracting the immunosuppressive effects of the tumor microenvironment, are currently being explored. The growing number of clinical trials evaluating immunotherapeutic agents in BTCs suggests that the incorporation of advanced genomic and transcriptomic profiling, including scRNA-seq, may facilitate the identification of patient subgroups most likely to benefit from immune-targeting therapies. As research advances, comprehending the intricate interactions among immune checkpoints, tumor mutational burden, and the tumor microenvironment will be crucial for enhancing immunotherapy strategies in iCCA and other biliary tract cancers, offering renewed prospects for patients with restricted treatment alternatives.

## 7 GENETIC PREDISPOSITION AND HEREDITARY FACTORS

### 7.1 Familial ICC and Potential Hereditary Syndromes

Although the majority of iCCA cases are sporadic, familial iCCA and its correlation with certain genetic disorders are gaining recognition. Genetic predisposition contributes to a minority of instances, frequently associated with hereditary mutations that increase susceptibility to cancer growth. Hereditary cancer syndromes, including Lynch syndrome [related to mutations in mismatch repair (MMR) genes] and Li-Fraumeni syndrome (connected to *TP53* mutations), may elevate the chance of developing iCCA. Furthermore, hereditary mutations in BRCA1/2, typically linked to breast and ovarian cancers, have been connected with certain instances of cholangiocarcinoma, indicating a wider involvement in cancer susceptibility. Other familial disorders, including hereditary hemochromatosis and primary sclerosing cholangitis (PSC), may elevate the risk of iCCA due to persistent inflammation and hepatic injury [95]. Comprehending these hereditary connections is essential for early identification, genetic counseling, and customized monitoring measures in high-risk families. Recent genetic data, particularly from the multi-omics study, have yielded essential insights into the invasive characteristics of ICC, particularly CA19-9-positive tumors [108]. The work integrated data from various clinical cohorts, synthesizing findings from whole-exome sequencing, transcriptomics, proteomics, single-cell RNA sequencing, and spatial transcriptomics to provide a comprehensive understanding of intrahepatic cholangiocarcinoma biology. The results indicated that CA19-9-positive ICCs exhibit significantly greater aggressiveness, with substantially reduced overall survival (median 24.1 months) and recurrence-free survival (median 11.7 months) in comparison to CA19-9-negative cases. Genetically, CA19-9-positive tumors demonstrated an increased incidence of KRAS mutations, whereas IDH1/2 mutations were more common in the CA19-9-negative cohort. Transcriptomic profiling indicated an increase in glycolysis-related pathways, implying a metabolic change that facilitates tumor development. Single-cell analysis revealed different cellular subpopulations, Epi_SLC2A1, CAF_VEGFA, and Mph_SPP1, linked to hypoxia-induced metabolic reprogramming. These subclusters established interactive cellular communities that facilitated epithelial-mesenchymal transition (EMT) and angiogenesis, both of which are critical characteristics of tumor invasiveness. The study discovered six possible therapeutic molecules through drug sensitivity analysis, presenting potential options for focused treatment. This integrative approach may enhance our comprehension of ICC heterogeneity and emphasize the clinical significance of CA19-9 as a prognostic biomarker. The results can facilitate the development of more tailored and efficacious treatment approaches for this challenging cancer.

### 7.2 Role of Environment-Genetic Interactions

Environment-genetic interactions significantly influence the development of iCCA. Environmental variables, including chronic liver inflammation, parasite infections (*e.g*., *Opisthorchis viverrini*), hepatitis B and C infections, and exposure to chemicals, such as thorotrast or dioxins, can induce genetic changes that facilitate carcinogenesis [96]. Individuals possessing predisposing genetic variants, such as those in *TP53*, *IDH1*/2, or *FGFR2*, may exhibit increased susceptibility to certain environmental triggers. Moreover, persistent inflammation cultivates a mutagenic milieu, heightening the probability of DNA damage and epigenetic modifications. Comprehending these connections is crucial for risk evaluation, early identification, and preventive measures in iCCA.

### 7.3 Impact of Liver Diseases (*e.g*., Cirrhosis, Viral Hepatitis) on Genetic Mutation Susceptibility

Chronic liver illnesses, including cirrhosis and viral hepatitis (HBV, HCV), markedly elevate the risk of genetic alterations that promote the development of iCCA [97]. Chronic liver inflammation and fibrosis establish a pro-mutagenic milieu by producing oxidative stress, causing DNA damage, and hindering DNA repair processes. This facilitates mutations in critical oncogenes and tumor suppressor genes, such as *TP53*, *KRAS*, and *IDH1*/2. The hepatitis B virus (HBV) can incorporate its DNA into the host genome, immediately inducing genetic instability, whereas the hepatitis C virus (HCV) instigates chronic inflammation. These hepatic disorders not only heighten sensitivity to mutations, but also facilitate tumor advancement and therapeutic resistance.

## 8 GENETIC ENGINEERING AND MODEL SYSTEMS FOR ICC

### 8.1 Use of CRISPR/Cas9 for Modeling Genetic Alterations in ICC

CRISPR/Cas9 technology has emerged as a potent tool for simulating genetic modifications in iCCA, yielding significant insights into tumor biology and therapeutic advancement. CRISPR/Cas9 facilitates accurate genome editing, enabling researchers to induce specific mutations, like those in *FGFR2*, *IDH1*/2, and *TP53*, frequently linked to iCCA. This establishes precise *in vitro* and *in vivo* models that replicate the genetic profile of iCCA, facilitating the investigation of carcinogenesis, the identification of prospective therapeutic targets, and the evaluation of therapy responses [98]. Researchers can utilize *CRISPR/Cas9* to create *IDH1* mutations or *FGFR2* fusions in hepatocyte or cholangiocyte cell lines, facilitating the investigation of how these modifications influence tumor initiation, development, and metastasis [99]. Moreover, these models enable the assessment of targeted medicines, such as FGFR inhibitors and *IDH* inhibitors, to determine their efficacy in a genetically pertinent setting.*CRISPR/Cas9* models can enhance the comprehension of iCCA and expedite the identification of innovative treatment approaches.

### 8.2 Animal Models for Studying Genetic Mutations

Animal models play a crucial role in studying genetic mutations in iCCA, helping researchers understand tumorigenesis and test therapeutic strategies. Genetically engineered mouse models (GEMMs) allow for the introduction of specific mutations, such as those in *IDH1*/2, *TP53*, or *FGFR2*, commonly seen in iCCA [100]. These models closely mimic human iCCA's genetic landscape and tumor progression, providing insights into the molecular mechanisms driving the disease. Additionally, xenograft models, where human iCCA cells are implanted into immunocompromised mice, enable the evaluation of tumor growth and response to therapies. These models are also valuable for testing the efficacy of targeted treatments, such as *IDH* inhibitors or FGFR inhibitors. Overall, animal models provide essential tools for exploring genetic alterations in iCCA, facilitating the development of novel therapeutic approaches and improving our understanding of the disease's biology.

### 8.3 Patient-derived Organoids and Xenograft Models for Exploring ICC Genetics

Patient-derived organoids and xenograft models are potent instruments for investigating the genetics of iCCA. Organoids, originating from patient tumor tissue, replicate the architectural and genetic characteristics of the original tumor, facilitating individualized investigations of iCCA's genetic alterations and therapy responses [101]. Xenograft models, involving the implantation of human tumor cells into immunocompromised mice, enable researchers to monitor tumor proliferation and evaluate therapeutic approaches *in vivo*. Both models elucidate the genetic modifications propelling iCCA, including *FGFR2* fusions and *IDH* mutations, and are crucial for formulating targeted therapeutics and comprehending tumor dynamics.

## 9 CHALLENGES AND FUTURE DIRECTIONS IN GENETIC RESEARCH FOCUSED ON ICC

### 9.1 Current Limitations in Understanding ICC Genetics

Despite progress in comprehending the genetics of iCCA, numerous limits persist. The variability of iCCA hinders the discovery of universal genetic causes, as tumors may display various mutations and molecular profiles. Interactions within the tumor microenvironment and epigenetic changes further complicate genetic understanding. Moreover, insufficient clinical evidence from varied populations restricts the extrapolation of results. Inconsistent presence of rare genetic alterations, including *FGFR2* fusions and *IDH* mutations, complicates the formulation of comprehensive diagnostic and treatment solutions [102]. These challenges highlight the necessity for a comprehensive, interdisciplinary study to thoroughly comprehend iCCA genetics. iCCA studies are limited by tumor heterogeneity, small sample sizes, geographic variability, and differing molecular subtypes. Immunotherapy response varies due to distinct immune landscapes. Data standardization challenges affect reproducibility, and findings may not fully apply across diverse patient populations or treatment settings.

### 9.2 Emerging Technologies in Genetic Research (*e.g*., Next-generation Sequencing)

Innovative technologies, especially next-generation sequencing (NGS), are revolutionizing genetic research in iCCA. NGS facilitates high-throughput, accurate examination of complete genomes, exomes, or targeted gene panels, elucidating a comprehensive genetic profile of iCCA. This method enables researchers to pinpoint essential mutations and modifications, including *FGFR2* fusions, *IDH1*/2 mutations, *TP53* mutations, and *KRAS* mutations, which are vital for comprehending tumor initiation, development, and therapy resistance. Furthermore, single-cell RNA sequencing facilitates the examination of gene expression profiles at the individual cell level, yielding enhanced understanding of tumor heterogeneity, cancer stem cells, and immune cell interactions within the tumor microenvironment [103]. This method assists in identifying cell subpopulations with unique genetic profiles that may contribute to metastasis or therapeutic resistance. Liquid biopsy, which examines circulating tumor DNA (ctDNA) from blood or other physiological fluids, is an emerging method that facilitates non-invasive monitoring of genetic changes and tumor progression. Liquid biopsies can identify early recurrence, detect resistance mutations, and monitor therapeutic response in real-time, providing a dynamic method for disease management. Collectively, these sophisticated technologies are enhancing our comprehension of iCCA genetics, facilitating individualized treatment approaches, augmenting early detection, and promoting the advancement of targeted treatments and diagnostic biomarkers. Single-cell RNA sequencing (scRNA-seq) has revolutionized cancer research by revealing tumor heterogeneity, cancer stem cells (CSCs), and immune interactions at the level of individual cells. This study identifies various subpopulations within tumors, each exhibiting unique gene expression profiles and mechanisms of resistance to therapy. Cancer stem cells, identified *via* single-cell RNA sequencing, are instrumental in metastasis and relapse, offering distinct therapeutic targets. The tumor microenvironment, comprising immune cells, fibroblasts, and endothelial cells, is analyzed through scRNA-seq to elucidate immune evasion strategies. Single-cell RNA sequencing in CCA provides enhanced understanding of tumor evolution, emphasizing immune suppressive pathways and identifying potential biomarkers for precision therapies. The integration of scRNA-seq with multi-omics has the potential to enhance treatment strategies [104].

### 9.3 Potential for Personalized Medicine and Genetic-based Prevention Strategies

Personalized medicine presents considerable promise in the treatment of iCCA, facilitating customized therapeutic approaches informed by the genetic profiles of individual patients. By identifying particular genetic mutations, like *FGFR2* fusions, *IDH1*/2 mutations, *TP53* mutations, and *KRAS* mutations, clinicians can choose tailored medications that are more efficacious and less harmful than traditional treatments [57]. FGFR inhibitors and *IDH* inhibitors have demonstrated encouraging outcomes in clinical trials for patients with relevant genetic mutations. This method enhances treatment results while reducing unwanted effects, providing a more tailored and effective approach to managing iCCA [105]. Alongside targeted therapies, genetic preventative measures can be formulated for those at elevated genetic risk of iCCA, particularly those with hereditary mutations or a familial history of liver conditions, such as primary sclerosing cholangitis (PSC) [106]. Genomic screening can identify high-risk patients who may benefit from early intervention, ongoing surveillance, and preventative strategies, like lifestyle adjustments or chemoprevention [107]. Furthermore, the advancement of liquid biopsies and other non-invasive genetic testing techniques enables real-time surveillance of tumor progression and therapeutic response, hence allowing for prompt modifications to treatment strategies. Ultimately, tailored medicine and genetic-based preventative techniques possess the capacity to enhance survival rates, diminish treatment-related toxicities, and optimize care for iCCA patients.

## CONCLUSION

In conclusion, advancements in genetic research and novel technologies, such as NGS, scRNA-seq, and liquid biopsies, are enhancing our understanding of iCCA. These advancements provide an enhanced understanding of tumor heterogeneity, clonal evolution, and resistance mechanisms, facilitating the formulation of more precise and effective treatment strategies. Genetic profiling has transformed personalized medicine by enabling clinicians to customize therapies according to specific genomic mutations, including *FGFR2* fusions, *IDH1/IDH2* mutations, and *BAP1* alterations. Targeted therapies based on genomic insights enhance treatment efficacy and reduce toxicity, thereby improving patient quality of life. In addition to optimizing treatment, genetic-based preventive strategies offer significant potential for risk stratification and early intervention. Identifying individuals with genetic markers or familial risk factors enables clinicians to implement proactive surveillance strategies, potentially facilitating earlier detection and enhancing prognosis. Integrative multi-omics approaches are revealing new biomarkers that could function as potential therapeutic targets, facilitating advancements in precision oncology. Immunotherapy is increasingly prominent in iCCA research, with checkpoint inhibitors and combination strategies demonstrating potential, especially in tumors exhibiting microsatellite instability (MSI) or mismatch repair deficiency (dMMR). These advancements indicate a transition to a more personalized approach, wherein treatment decisions are informed by a thorough molecular analysis of each patient's tumor profile. Ongoing research necessitates collaborative international efforts and extensive genomic studies to enhance therapeutic interventions and optimize patient outcomes. The future of iCCA treatment is progressing towards a precision-based approach, incorporating molecular diagnostics, targeted therapies, and immunomodulation, with the goal of improving survival rates and overall quality of life for affected individuals.

## LIST OF ABBREVIATIONS

CAFs: Cancer-associated Fibroblasts
CCA: Cholangiocarcinoma
CEA: Carcinoembryonic Antigen
CSCs: Cancer Stem Cells
CT: Computed Tomography
ctDNA: circulating tumor DNA
dCCA: Distal Cholangiocarcinoma
dMMR: deficient Mismatch Repair
ECC: Extrahepatic Cholangiocarcinoma
ECM: Extracellular Matrix
EMT: Epithelial-to-mesenchymal Transition
FGFRs: Fibroblast Growth Factor Receptors
GEMMs: Genetically Engineered Mouse Models
HGF: Hepatocyte Growth Factor
HIFs: Hypoxia-inducible Factors
iCCA: Intrahepatic cholangiocarcinoma
ICIs: Immune Checkpoint Inhibitors
LINEs: Long Interspersed Nuclear Elements
MDSCs: Myeloid-derived Suppressor Cells
MMPs: Matrix Metalloproteinases
MMR: Mismatch Repair
MRI: Magnetic Resonance Imaging
MSI: Microsatellite Instability
NAFLD: Non-alcoholic Fatty Liver Disease
NGS: Next-generation Sequencing
pCCA: Perihilar Cholangiocarcinoma
PSC: Primary Sclerosing Cholangitis
RTKs: Receptor Tyrosine Kinases
TAMs: Tumor-associated Macrophages
TCGA: The Cancer Genome Atlas
TMB: Tumor Mutational Burden
TME: Tumor Microenvironment
WES: Whole-exome Sequencing
WGBS: Whole-genome Bisulfite Sequencing
WGS: Whole-genome Sequencing
